# Identification of novel cell-free RNAs in maternal plasma as preterm biomarkers in combination with placental RNA profiles

**DOI:** 10.1186/s12967-023-04083-w

**Published:** 2023-04-12

**Authors:** Heyue Jin, Yimin Zhang, Zhigang Fan, Xianyan Wang, Chen Rui, Shaozhen Xing, Hongmei Dong, Qunan Wang, Fangbiao Tao, Yumin Zhu

**Affiliations:** 1 Department of Maternal, Child and Adolescent Health, School of Public Health, Anhui Medical University, No 81 Meishan Road, Hefei, Anhui China; 2 MOE Key Laboratory of Population Health Across Life Cycle, No 81 Meishan Road, Hefei, Anhui China; 3 Anhui Provincial Key Laboratory of Population Health and Aristogenics, Anhui Medical University, No 81 Meishan Road, Hefei, Anhui China; 4 NHC Key Laboratory of Study on Abnormal Gametes and Reproductive Tract, Anhui Medical University, Hefei, Anhui China; 5 Department of Neonatology, Ma’anshan Maternal and Child Health Hospital, Ma’anshan, Anhui China; 6 Department of Toxicology, School of Public Health, Anhui Medical University, Hefei, Anhui China; 7 Key Laboratory of Environmental Toxicology of Anhui Higher Education Institutes, Hefei, Anhui China; 8 MOE Key Laboratory of Bioinformatics, Center for Synthetic and Systems Biology, School of Life Sciences, Tsinghua University, Beijing, China; 9 Department of Obstetrics, Ma’anshan Maternal and Child Health Hospital, Ma’anshan, Anhui China

**Keywords:** Preterm birth, Prediction, Cell-free RNA, Placenta, Plasma, Transcriptome

## Abstract

**Background:**

Preterm birth (PTB) is the main driver of newborn deaths. The identification of pregnancies at risk of PTB remains challenging, as the incomplete understanding of molecular mechanisms associated with PTB. Although several transcriptome studies have been done on the placenta and plasma from PTB women, a comprehensive description of the RNA profiles from plasma and placenta associated with PTB remains lacking.

**Methods:**

Candidate markers with consistent trends in the placenta and plasma were identified by implementing differential expression analysis using placental tissue and maternal plasma RNA-seq datasets, and then validated by RT-qPCR in an independent cohort. In combination with bioinformatics analysis tools, we set up two protein–protein interaction networks of the significant PTB-related modules. The support vector machine (SVM) model was used to verify the prediction potential of cell free RNAs (cfRNAs) in plasma for PTB and late PTB.

**Results:**

We identified 15 genes with consistent regulatory trends in placenta and plasma of PTB while the full term birth (FTB) acts as a control. Subsequently, we verified seven cfRNAs in an independent cohort by RT-qPCR in maternal plasma. The cfRNA *ARHGEF28* showed consistence in the experimental validation and performed excellently in prediction of PTB in the model. The AUC achieved 0.990 for whole PTB and 0.986 for late PTB.

**Conclusions:**

In a comparison of PTB versus FTB, the combined investigation of placental and plasma RNA profiles has shown a further understanding of the mechanism of PTB. Then, the cfRNA identified has the capacity of predicting whole PTB and late PTB.

**Supplementary Information:**

The online version contains supplementary material available at 10.1186/s12967-023-04083-w.

## Introduction

Preterm birth (PTB) is the leading cause of death in children under 5 years old worldwide. It is estimated that 17.7% of global under-five child deaths and 36.1% of 0–27 day neonatal deaths were due to complications from PTB [1]. In addition, PTB is the leading risk factor that contributes to growth disorders such as cognitive, visual, and learning disabilities [2]. Efforts to reduce both the incidence and mortality of PTB are still crucial [3].

Therefore, the development of predictive tools for identifying the risk of PTB from the antenatal population is of clinical relevance. A newly developed cervical elastography technique has been proposed for screening spontaneous preterm birth [4]. However, this method has not been used widely for early prediction of PTB because standardized baseline values for elastography parameters have not been established [4]. Recent studies have informed the characterization of a wide range of biological changes during pregnancy can be measured by plasma cell-free RNA (cfRNA) transcripts [5], plasma proteome [6], metabolomics [7–9], immunome [8], and microbiome [10, 11]. However, the prediction of PTB based on such molecular profiles is still challenging.

During pregnancy, the placenta is an important organ that connects the mother and the fetus. A placenta with impaired function may lead to reduced blood flow, or the transfer of oxygen and nutrient to the fetus, which could affect the growth and development of the fetus [12]. A recently published study provides a comprehensive assessment of alterations in the placental transcriptome correlated with spontaneous preterm birth, which suggested that we can obtain novel insight into the mechanisms of PTB by discriminating molecular differences in the placenta [13]. The cfRNA is a new class of biomarkers with enormous potential for the non-invasive diagnosis, prognosis and surveillance in many diseases [14], which has been proven that released from maternal, fetal and placental tissue and can be detected in plasma after entering maternal bloodstream [15, 16]. Notably, placental development is closely linked to pregnancy health. The expression level of RNA measurement in maternal plasma may be a useful and convenient approach for reflecting placental gene-expression profiles as previously described. Thus, analysis of plasma RNA may also have critical significance in tracking pregnancy progression and fetal health [17, 18].

To consolidate the abovementioned considerations, our study tried to combine the placental RNA profiles and comprehensively explored the significance of plasma RNA signature in PTB. To evaluate the correlation of RNA expression regulation in plasma and placenta compared with PTB, we integrated a total of 62 RNA-seq datasets from the placenta and 15 cfRNA-seq datasets from the maternal plasma. Subsequently, we aimed to assess whether these cfRNAs with consistent expression regulation trends can serve as early biomarkers of PTB. Overall, our study offers the novel PTB biomarkers with the clinical significance and further elucidate that the regulation of RNA in the placenta can be revealed from the plasma cfRNA of pregnant women.

## Methods

### Study design and cohort

Placental samples for 31 PTB infants and 31 paired full term birth (FTB) infants were collected from the Ma’anshan Birth Cohort (MABC) study. We obtained transcriptome data by bulk RNA sequencing from these 62 samples. The plasma cfRNA-seq data were downloaded from Sequence Read Archive (SRA) database, including seven FTB samples and eight PTB samples (SRP130149). In addition, from March to June 2022, we collected 41 maternal plasma samples with recruitment criteria for singleton pregnancy live births as a validation cohort in Ma’anshan Maternal and Child Health Hospital for experimental verification of the identified changes in cfRNA expression. Informed consent was obtained from each participant. The maternal characteristics of participants are presented in Additional file 1: Table S1.

### Sample collection

For placenta tissues collected from the MABC study, a piece of placental lobule tissue was separated from the maternal side of the placenta at a distance of 5 cm from the umbilical cord within 30 min of delivery of the placenta. Each piece of tissue, about 1cm^3^ in volume, was placed in a cryostorage tube with an RNA later and refrigerated overnight at 4 °C, and then stored at − 80 °C after the RNA later was absorbed. For plasma samples collected from the validation cohort, based on an estimated due date from the last menstrual period (LMP), the samples of blood were collected before delivery at 37 weeks for the validation cohort. All samples were placed in EDTA tubes. Within 8 h of sample collection, the samples were centrifuged at 3000×*g* for 6 min to separate plasma and then reposited at − 80 °C until assay.

### RT-qPCR validation

CfRNAs were extracted from 0.8 ml plasma using the Trizol reagent (Invitrogen, USA). 10.5 µl RNA was reverse transcribed using First Strand cDNA Synthesis Kit (Promega, USA) with adding 1 µl External RNA Controls Consortium (ERCC) according to the manufacturer’s protocol. The cDNA was diluted twice and amplified by SYBR green (YESEN, Shanghai, China) in a LightCycler® 96 System (Roche).

Glyceraldehyde-3-phosphate dehydrogenase (GAPDH) is the internal reference for cfRNAs. Primers for each differentially expressed gene were designed by an online database PrimerBank and Integrative Genomics Viewer (IGV) [19, 20]. All sequences are enumerated in Additional file 1: Table S2. The average of the three replicates was performed as the cycle threshold (Ct) value for each cfRNA. Several cfRNAs which considered undetected on the basis of the Ct value limit of 39 were excluded for further analysis [21]. For the evaluation of cfRNA expression levels, we used the 2-ΔCt method and then normalized.

### Differential expression analysis

Differentially expressed genes (DEGs) between the PTB cases and controls were identified using edgeR [22]. The significance cutoff was appraised at |log_2_(fold-change)| ≥ 0.59 and p-value < 0.05. All information of DEGs was used for principal component analysis (PCA) in each group independently. The comparison of DEGs was performed by the “UpSetR” R package.

### Functional enrichment analysis

We applied g: Profiler for performing Gene Ontology (GO) and Kyoto Encyclopedia of Genes and Genomes (KEGG) analysis [23]. The results of enrichment pathways were ranked based on adjusted p-value and plotted in R statistical software.

### Protein–protein interaction (PPI) network

We respectively incorporate genes with altered expression levels in plasma and the placenta in PTB to construct the protein–protein interaction (PPI) network. The PPI network was implemented using the STRING database and then the key nodes were screened using the cytohubba in Cytoscape software with four methods including Maximal Clique Centrality (MCC), Maximum Neighborhood Component (MNC), degree, and closeness [24, 25]. The top 25 genes were selected as the key genes in each algorithm based on the score. We identified the final hub genes by intersecting the key genes obtained by these algorithms [26]. Metascape provides the functional annotation of these hub genes [27].

### Establishment of a predictive model

To predict PTB (GA at delivery < 37 weeks), we developed a machine learning model of support vector machine (SVM), which relying on a dataset of the measured relative expression levels of cfRNAs by RT-qPCR. The expression levels of cfRNAs whose results of RT-qPCR were consistent with RNA-seq results and showed significantly difference in the preterm and term groups were perceived as input features. All samples we collected were divided into 50% train set and 50% test set by the createDataPartition method from the caret package in R statistical software. The area under the computing receiver-operating characteristic (ROC) curve (AUC) was calculated to assess the performance of the machine learning model. In addition, maternal age, parity, body-mass index (BMI), platelet distribution width (PDW), neutrophil-to-lymphocyte ratio (NLR), and haemoglobin (HB) were considered as preterm clinical risk factors through literature searching [28–33]. Then, we collected the above clinical data matched to samples used for independent validation by RT-qPCR through electronic medical records.

### Statistical analysis

A nonparametric Mann–Whitney U-test was used for all tests designed to compare the expression level of different groups. The significant differences were considered p-value < 0.05.

## Results

### Discovery of distinct dysregulated genes in RNA-seq data and the pathways involved in preterm birth

**Fig. 1:**
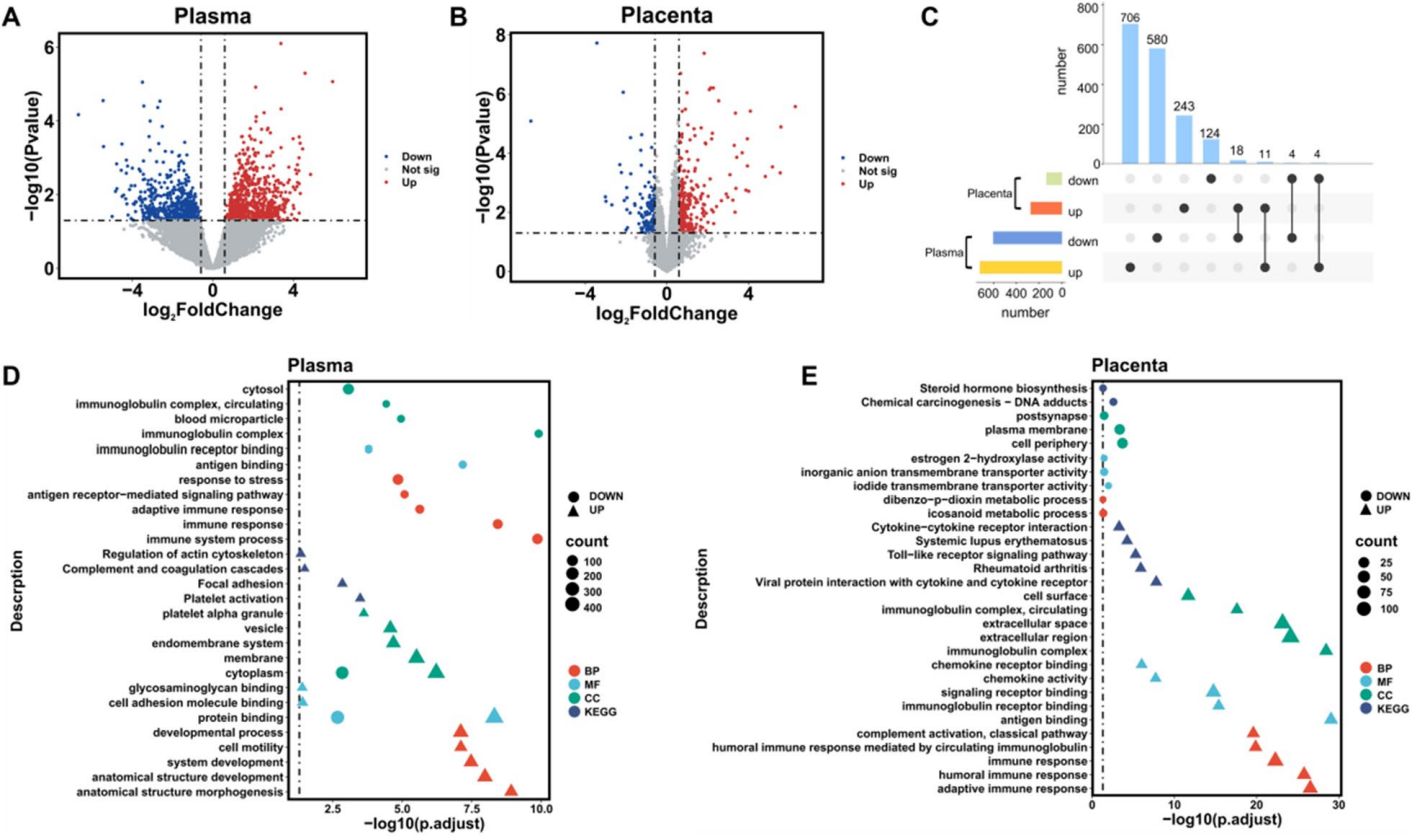
Differentially expressed genes in preterm birth placenta and plasma. **A** Volcano plots of differentially expressed genes (DEGs) in the comparison (PTB vs. FTB) of RNA-seq data from plasma. **B** Volcano plots of differentially expressed genes (DEGs) in the comparison (PTB vs. FTB) of RNA-seq data from placenta. **C** UpSet plot of the gene symbol mapping overlaps for all sets of DEGs’ comparisons. **D** The top five GO-terms and KEGG pathways for the enrichment of DEGs involved in plasma group. **E** The top five GO-terms and KEGG pathways for the enrichment of DEGs involved in placenta group

We first identified DEGs by analyzing cfRNA-seq in plasma, including a binary grouping of seven PTB (< 37 weeks) versus eight FTB (≥ 37 weeks). Additionally, we applied PCA to our placental and plasma dataset, to explore sample features at gene expression levels (Additional file 1: Fig. S1A, B). The FTB samples were mixed with PTB samples, which may be caused by large heterogeneity. Then we observed broad differences using the FTB group as control with 721 up-regulated genes and 602 down-regulated genes in plasma (Fig. 1A). For the placental transcriptome, we only identified 404 DEGs by comparing 31 PTB samples with 31 FTB samples using tissue RNA-seq (Fig. 1B). We found 11 RNAs were up-regulated both in plasma and placenta of PTB group, which accounted for 1.5% and 4.0% of up-regulated genes in plasma and placenta, respectively (Fig. 1C, and Additional file 1: Fig. S2A); and 4 RNAs were down-regulated both in plasma and placenta of PTB group, which accounted for 0.66% and 3.0% of down-regulated genes in plasma and placenta, respectively (Fig. 1C, and Additional file 1: Fig. S2A).

GO enrichment and pathway analyses were using differently expressed gene-set and the top five pathways in each group measured by adjusted p-value were presented. We found that the DEGs in plasma were involved in distinct biological process (BP) terms from those in placenta. The up-regulated genes in PTB in plasma were mainly involved in developmental and cellular process (Fig. 1D). By contrast, the most significant enrichment pathway of up-regulated genes in PTB in the placenta that was individually involved with immune response (Fig. 1E). The down-regulated genes in PTB in plasma showed the relationship with immune pathways, whereas most down-regulated genes in PTB in the placenta were associated with metabolic process (Fig. 1D, E). We detected significant enrichment of DEGs of plasma in most molecular function (MF) terms related to binding, such as protein binding, glycosaminoglycan binding, and immunoglobulin receptor binding (Fig. 1D). In the placenta, the enrichment analysis showed that these up-regulated genes were also enriched in signaling receptor binding, and the down-regulated genes were mainly enriched in transmembrane transporter activities (Fig. 1E). AS for the enrichment result of up-regulated genes in plasma, the top five significant cell component (CC) terms were dominated by membrane-bounded organelle and endomembrane system, while the down-regulated genes related to the immunoglobulin complex and intracellular anatomical structure (Fig. 1D). In the placenta, the majority of genes associated with the extracellular region and immunoglobulin complex pathway were up-regulated in PTB (Fig. 1E). Cell periphery was also amongst the significant pathways in down-regulated genes in PTB.

We further performed KEGG pathway analysis for plasma and placental DEGs respectively. Only four KEGG pathways were detected in plasma group, including regulation of actin cytoskeleton, complement and coagulation cascades, focal adhesion, and platelet activation (Fig. 1D). These representative entries of the analysis of placental-derived DEGs mostly contained human disease, steroidogenesis and signaling interaction (Fig. 1E). In total, the immune signaling subgroup was the largest enriched, which consistent with the previous finding on the pathologic mechanisms of PTB [34]. When we compared the pathway enrichment of up-regulated genes in plasma vs. the up-regulated genes in the placenta, 23 common pathways were revealed which accounted for a higher proportion of all plasma and placental pathways (Additional file 1: Fig. S2A). The data reveal that up-regulated genes in PTB have a distinct association in the response to stimulus and glycosaminoglycan binding for the BP and MF term while sharing enrichment in CC related to the extracellular region (Additional file 1: Fig. S2C). The same pathway both in plasma and placenta has not been identified in the down-regulated genes enrichment pathway.

### The complexity of RNA regulatory molecules in plasma and placenta

**Fig. 2:**
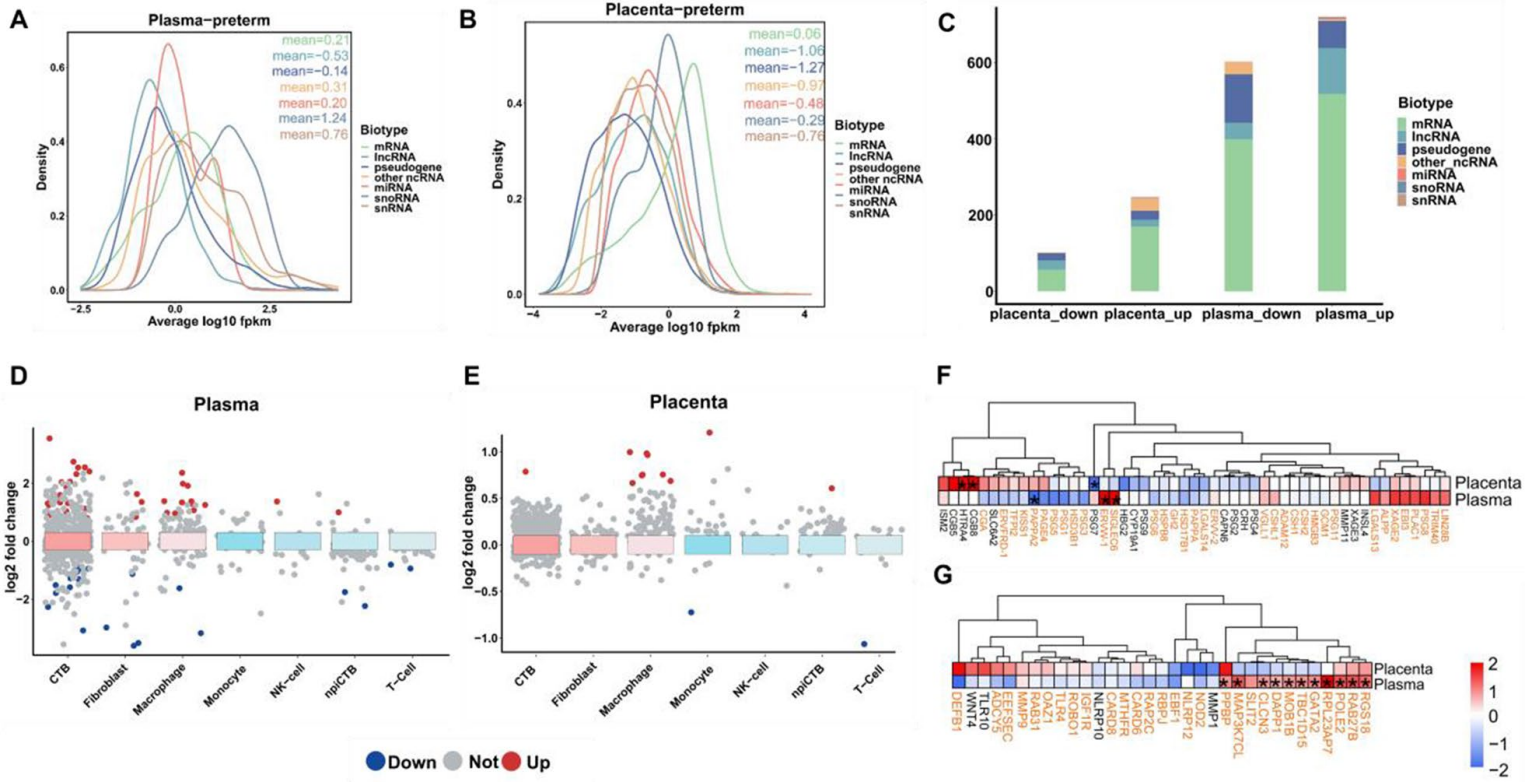
Assessment of RNA regulatory molecular features detected in plasma and placental transcriptomes. **A**, **B** The density of expression abundance in preterm birth of different RNA biotypes compared in **A** plasma, and **B** placenta. **C** Numbers of differently expressed RNAs for different RNA biotypes. **D** Several placental cell type specific genes were differentially expressed in PTB in the maternal plasma cfRNA profile. **E** Several placental cell type specific genes were differentially expressed in PTB in the placental RNA profile. **F** The fold change of placenta-associated genes between PTB and FTB pregnancies. **G** The fold change of PTB-associated genes between PTB and FTB pregnancies. The orange indicates that the value of |log_2_ fold change| is higher in plasma, compared to the placenta group. *p-value < 0.05

We separately observed the distribution of expression levels of different biotypes of RNA molecules co-detected from plasma and placenta. The expression levels of the different RNA classes in the PTB group were skewed between maternal plasma and the placenta. We found that the majority of annotated mRNA, other non-coding RNAs (ncRNAs), snoRNAs, and snRNAs were expressed weakly in the PTB placenta compared with the PTB plasma (Fig. 2A, B). By contrast, the expression levels of long noncoding RNAs (lncRNAs) and pseudogenes in placenta is higher than that in the plasma for the PTB group (Fig. 2A, B). We also obtained this similar tendency in the FTB group except for the pseudogenes (Additional file 1: Fig. S3A, B). Subsequently, we ranked the total number of RNA biotypes that were involved in the aberrant regulation of placental and plasma respectively (Fig. 2C). Consistent with the plasma, we found that the majority of DEGs in the placenta were distributed in the following types: mRNA, lncRNA, and pseudogene (Fig. 2C).

Previous work in humans has confirmed that single-cell resolution (scRNA-seq) can catch a wide range of cell types that contribute to the placenta and identified distinct differences in the cell type components of preterm and full-term pregnancies [35, 36]. To further evaluate the alterations in gene expression in PTB, we attempted to map placental and plasma DEGs to genes with altered expression in different placental cell subtypes and tested for the presence of single cell-derived placental profiles in cfRNA in the maternal circulation [35]. The major populations include cytotrophoblast (CTB), fibroblast, macrophage, monocyte, NK-cell, npiCTB, and T-cell. In the maternal plasma cfRNA profile, the largest number of DEGs between the PTB and FTB groups were observed in the CTB, followed by macrophage (Fig. 2D). Strikingly, the majority of cell marker genes were robustly increased in PTB. Using the placental profile, we found many of macrophage marker genes were significantly altered expression in PTB compared to the FTB group (Fig. 2E). These results validate the macrophage signature changes in preterm pregnancy that were previously reported, and further revealed the single-cell features can be detected non-invasively in the maternal circulation throughout the pregnancy period [35]. Then, we collected 33 PTB-associated genes from several studies, which were identified from different data types (ChIP-seq, RNA-seq, methylation, and others) [5, 37–42]. Meanwhile, 51 genes with differential expression in complicated pregnancies and reflection of placenta function development were defined as placenta-associated genes from a comprehensive study of the human placenta transcriptome [43]. Both placenta-associated genes and PTB-associated genes showed greater differences in plasma (PTB group vs. FTB group), indicating that plasma may amplify regulatory signals, and demonstrating that RNA signals in plasma can be used as markers to detect pregnancy status (Fig. 2F, G). In addition, we focused on the correlation between changes in the expression levels of each type of RNA in placental tissue and maternal plasma. Among them, the regulation trend of mRNA, snoRNA, and pseudogene reached a significant level (Additional file 1: Fig. S3C, E, G). By contrast, such significant correlation was lack for snRNA and lncRNA (Additional file 1: Fig. S3D, F).

### Construction of protein–protein interaction network and identification of hub genes

**Fig. 3:**
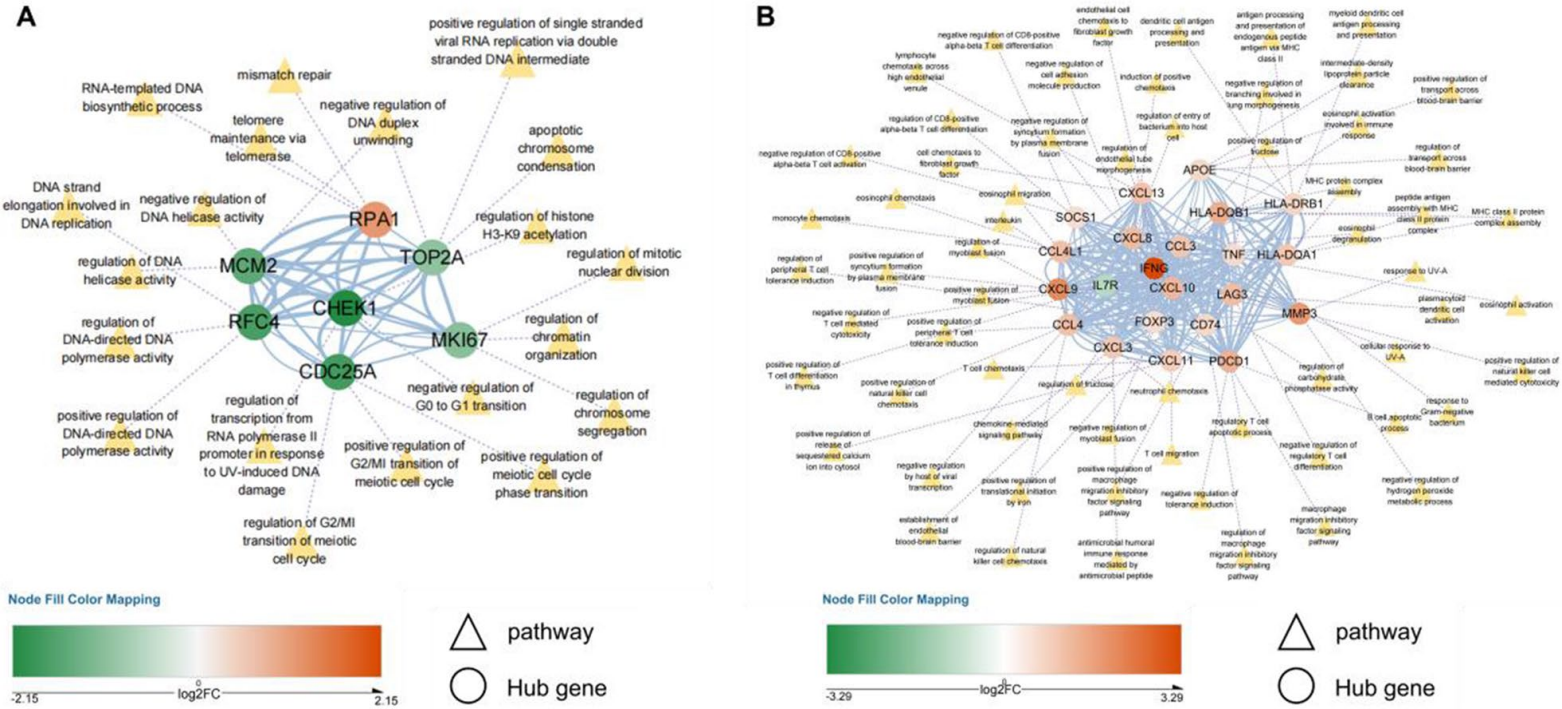
Selection of hub genes in protein–protein interaction (PPI) network. **A** The interaction diagram of PPI network by 7 hub genes from plasma. **B** The interaction diagram of PPI network by 22 hub genes from placenta. Network nodes and edges represent genes and gene–gene associations. Blue solid lines represent combination. Purple dotted lines represent the biological process terms corresponding to hub genes

Many proteins resulting from the disruption of molecular interaction networks are involved in the etiology of PTB [44]. The DEGs from the placenta and plasma were utilized to construct the PPI network. The PPI network was constructed using STRING and visualized using Cytoscape. We obtained a PPI network with 893 nodes and 2,403 edges using DEGs from plasma (Additional file 1: Fig. S4A). Another PPI network constructed by DEGs from the placenta has 225 nodes and 359 edges (Additional file 1: Fig. S4B). The degree, MNC, closeness, and MCC algorithms in the cytoHubba plugin were used to calculate the PPI network of these DEGs, and the top 25 genes were selected as the key genes (Additional file 1: Fig. S5A, B). In plasma and placenta samples, a total of 7 hub genes and 22 hub genes were respectively defined based on the intersection of these algorithms (Fig. 3A, B, Additional file 1: Fig. S5C). There was no overlap of hub genes in the plasma and placenta samples (Additional file 1: Fig. S5D). We also found a few overlapped genes in the plasma and placenta samples of the top 25 key genes identified by each algorithm (Additional file 1: Fig. S5D). Moreover, we found that hub genes from plasma are involved in nucleic acid metabolic process, protein modification process, and cell cycle phase transition (Fig. 3A, Additional file 2 : Table S3). Then we observed the hub genes from the placenta are related to the positive regulation of translation, cytokine-mediated signaling pathway, antigen processing and presentation, and cellular process primarily (Fig. 3B, Additional file 2 : Table S3).

### Identification of cfRNAs as potential biomarkers for preterm birth

**Fig. 4:**
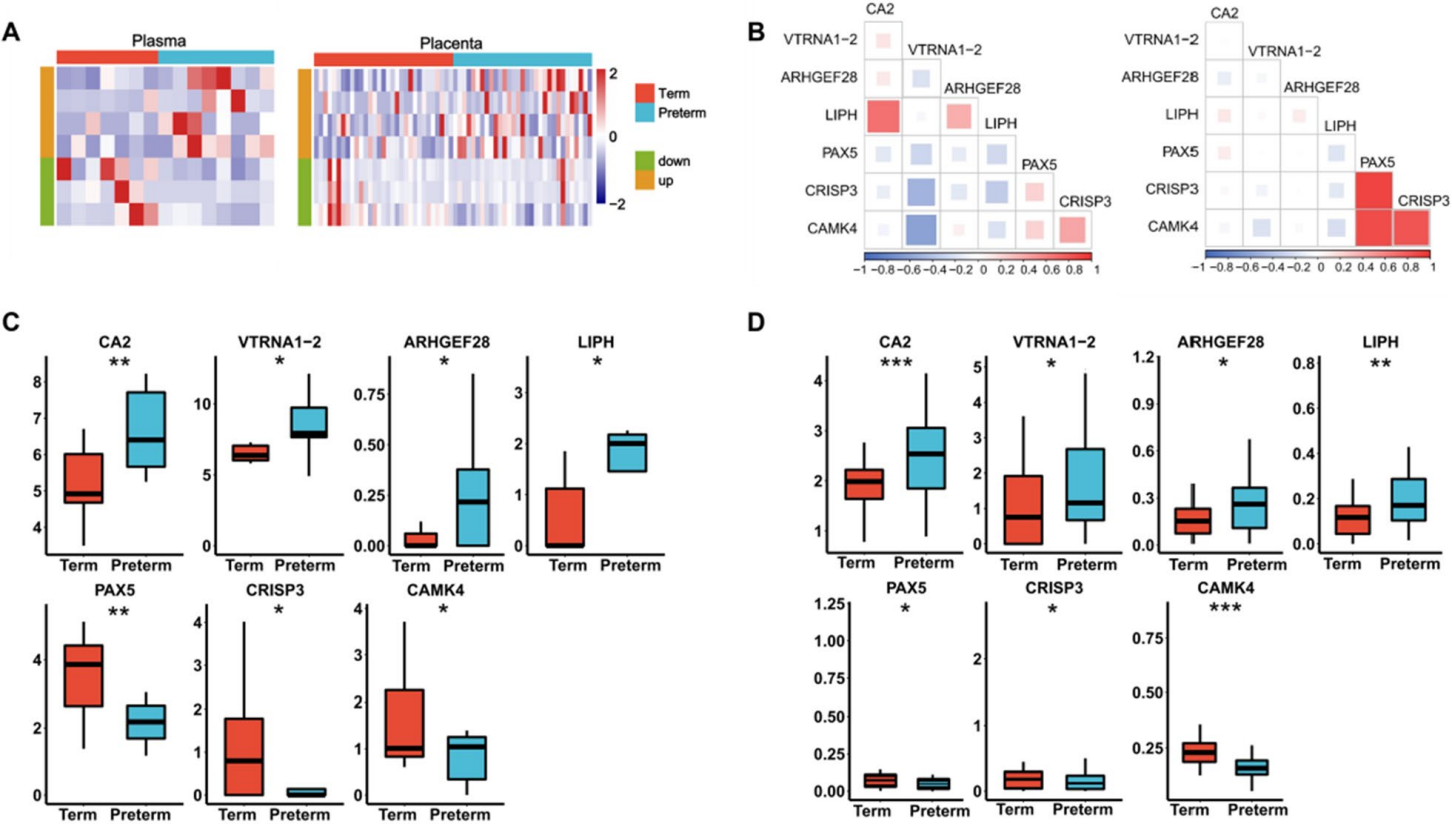
Candidate cfRNAs for predicting preterm birth. **A** Heatmap showing expression level in each samples of candidate genes in plasma and placental RNA-seq datasets (plasma samples: seven FTB vs. eight PTB, placenta samples: 31 FTB vs. 31 PTB). **B** Correlation heatmap displaying the inter connectivity among candidate genes. The size of the squares and the color scale correlate to the correlation of gene expression in RNA-seq data including plasma and placenta. left: plasma, right: placenta. **C** Expression level for differentially expressed genes in the discovery based on plasma cfRNA-seq datasets. **D** Expression level for differentially expressed genes in the discovery based on placental RNA-seq datasets. *p-value < 0.05, **p-value < 0.01, ***p-value < 0.001

We identified 15 PTB candidate markers with a consistent regulation trend in the placenta and plasma by integrating placental RNA-seq and plasma cfRNA-seq, which were progressively narrowed to a panel of seven cfRNAs with the following criteria (Fig. 4A): (1) the up-regulated gene were filtered about the median values in the PTB group (median > 1, quantified using fpkm), and (2) the results of the comparison of the median values between the PTB group and the FTB group were required to be consistent with the regulatory trend results obtained by edgeR. Taken together, we generated a candidate marker set of seven cfRNAs for subsequent validation.

Then we explored the co-expression signatures of these genes in the placenta and plasma using Pearson correlation coefficient, which suggested that the occurrence of the high synergy of these genes is skewed in the plasma transcriptome compared to the placental transcriptome (Fig. 4B). Two sets of moderately related genes were present in the data from plasma dataset (*LIPH* and *CA2*, *CAMK4* and *VTRNA1-2*, 0.5 ≤ |R| < 0.8, p-value < 0.05; Fig. 4B). In placenta dataset, we found a strong positive correlation between *PAX5* and the two genes (*CRISP3*, *CAMK4*), which revealed that they may play a role in similar biological processes (R > 0.8, p-value < 0.05; Fig. 4B). All of these seven cfRNAs had the same expression patterns in both sets of samples from the placenta and plasma RNA-seq data. Among them, four cfRNAs (*CA2*, *VTRNA1-2*, *ARHGEF28*, and *LIPH*) were observed to be up-regulated and three cfRNAs (*PAX5*, *CRISP3*, and *CAMK4*) were down-regulated in PTB group compared with FTB group (Fig. 4C, D).

### Independent validation of the selected cfRNA biomarkers for preterm birth and predictive modeling of PTB

**Fig. 5:**
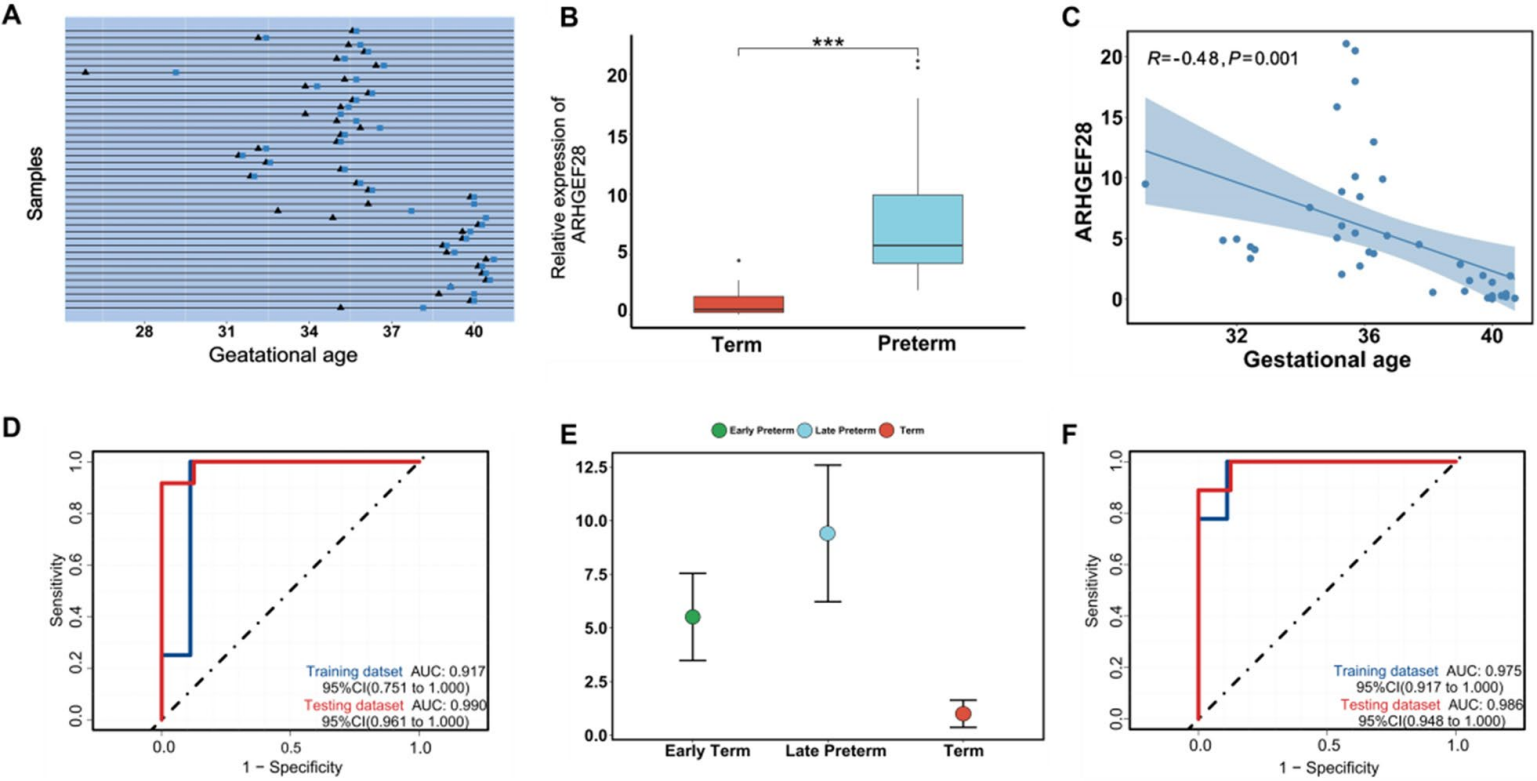
The validation of candidate markers and the performance of predictive models. **A** Blood sample collection timelines in the validation cohort. triangles: GA at blood collection, squares: GA at delivery. **B** Means ± SD for the relative expression level of *ARHGEF28* validated using RT-qPCR. ***p-value < 0.001 (Mann–Whitney U-test). **C** The correlation of the relative expression level of *ARHGEF28* with gestational age. **D** Receiver operating characteristic (ROC) curve representing prediction of preterm birth by the gene *ARHGEF28* across the training dataset and testing dataset using SVM algorithm. **E** Altered in the relative expression level of *ARHGEF28* in the three subgroups including early preterm, late preterm, and term. **F** ROC curves representing prediction of late preterm by the gene *ARHGEF28* across the training dataset and testing dataset using SVM algorithm

To further validate the potential PTB cfRNA biomarkers selected by the analyses described above, we used RT-qPCR for experimental verification in an independent cohort recruiting 41 plasma samples in mid to late pregnancy (Fig. 5A, Additional file 1: Table S1). In the PTB group, the 24 women delivered at 34.7 ± 1.9 weeks (average ± SD), while in the FTB group, the 17 women delivered at 39.7 ± 0.8 weeks. Based on the results of RT-qPCR, we found the *ARHGEF28* gene was significantly up-regulated in the plasma of PTB group, suggesting that *ARHGEF28* is a reliable biomarker for PTB prediction (Fig. 5B). In our study, most of FTB samples were collected later than PTB samples. Therefore, to investigate whether the expression level of *ARHGEF2*8 is altered with the progression of pregnancy, we built a subset of plasma samples collected at 35 ± 1 weeks and compared the expression level of *ARHEGF28* between preterm and full-term groups (13 PTB samples, 3 FTB samples). We found the *ARHGEF28* gene was still significantly up-regulated in PTB (Additional file 1: Fig. S6A), which suggested that the gene still has the potential to be a predictive biomarker for preterm birth if all sampling times are controlled to 35 ± 1 weeks. In addition, we tried to divide the 17 FTB samples into two groups according to the time point of collection (Before 37 weeks: n = 4, After 37 weeks: n = 13). Then we found no significant differences in the expression level changes of *ARHGEF28* between these two groups of samples (Additional file 1: Fig. S6B), which suggested that the gene may not be used as a biomarker for gestational development. Gestational age was considered to be a partial mediator between biological factors of PTB and neonatal outcomes, associated with neonatal morbidity in late preterm and early term birth [45]. Additionally, we also observed the significant correlation between gene expression level of *ARHGEF28* and gestational age: the decreased expression of *ARHGEF28* along with the gestational age increased gradually (p-value = 0.001, R = − 0.48) (Fig. 5C).

For the prediction of PTB, we divided the RT-qPCR data into training datasets with 21 samples and testing datasets with 20 samples (training dataset: 9 FTB samples, 12 PTB samples, testing dataset: 8 FTB samples, 12 PTB samples) at first. Next, we selected the cfRNA *ARHGEF28* as a feature applied to the PTB classifier. For the SVM model, the AUC was 0.917 in the training dataset and 0.990 in the testing dataset (Fig. 5D). Multiple clinical risk factors contribute to PTB, so we also collected other traditional risk factors that have been reported [28–33]. To assess the prediction capability for PTB of traditional risk factors, we integrated a base model depending on the following factors: maternal age, parity, BMI, PDW, NLR, HB. We presented the AUC of traditional risk factors with and without the addition of *ARHGEF28* applying the SVM machine model. After the exclusion of samples with missing clinical data, the training dataset included 19 samples, while the testing dataset included 17 samples. The lower AUC was yielded in the base model and then the AUC increased with the addition of *ARHGEF28* (Additional file 1: Fig. S7A–C). We calculated Pearson correlation coefficients to further assess the affiliation between the expression level of *ARHGEF28,* gestational age, and PTB-associated clinical characteristics, but no significant correlation was detected, which may cause by the small amount of clinical data (Additional file 1: Figs. S8A–F and S9A–F). Compared to the predictive model with *ARHGEF28* as an independent factor, the combination of *ARHGEF28* expression levels and clinical risk factors may have a more valid predictive performance by integrating more samples in the future work.

At last, we performed a separate analysis of PTB subgroups defined using the guideline from the American College of Obstetricians and Gynecologists (ACOG) [46]. The result reveals that there has been a rise in the relative expression level of *ARHGEF28* between the early-preterm group (28–34 weeks) and the late-preterm group (34–37 weeks), especially in the late-preterm group (Fig. 5E). We constructed a predictive model of late preterm using the similar approach, which still achieved an excellent performance (training dataset, AUC = 0.975; testing dataset, AUC = 0.986) (Fig. 5F). Late preterm is the largest group accounting for nearly three-fourths of all preterm infants, with the risk of increased neonatal morbidities as well as the risk of long-term adverse outcomes [47, 48]. Therefore, the detection of the risk of late preterm clinically remains necessary for infant development. The findings from our study confirmed the potential of *ARHGEF28* in the prediction of preterm birth and especially late preterm.

## Discussion

In this study, for the first time, we systematically delineated the expression profiles of aberrant regulatory genes associated with PTB in plasma and placenta, then identified a novel cfRNA as a biomarker for predicting PTB. By integrating and analyzing the RNA-seq data of placental tissue and the cfRNA-seq data of maternal plasma, we found that the differentially expressed RNA in placentas of preterm infants could be detected in the plasma of mothers. Although the main result here was the prediction of PTB, we further supported the altered expression level of genes in the placenta can be detected in maternal plasma. This also accords with the observations from earlier study which focused that the placental miRNA profiles combined with matched profiles from maternal plasma reflecting physiological changes occurring at early to middle gestation [49].

To explore the possible molecular mechanisms of PTB, we searched enriched pathways for the differentially expressed genes of PTB. More enrichment of immune pathways is in line with those of previous findings that pregnancy and parturition involve widespread changes in the maternal immune system [50]. Different from PTB-associated pathways in plasma, we detected distinct pathways in placenta group about metabolism. These are similarities between the observation described by a previous placental transcriptomic study of PTB [13]. Accumulating evidence indicates that abnormalities in maternal or fetal membrane metabolism stimulate inflammatory cytokines, which may drive PTB [51]. However, there were a few pathways associated with metabolic processes in our observations. The estrogen metabolism pathway, fetal stress, and fetal anomalies are the regularly reported pathways associated with PTB [51], although these pathways were not present in our result.

We next explored the regulation of PTB by different types of RNA molecules detected in placenta and plasma. Existing research revealed the critical association between placental gene expression levels and the abundance of the genes in maternal plasma [18]. We observed significant correlation between altered expression of mRNA and snoRNA in placenta and plasma in our data, this correlation pattern of mRNA is weak and the mismatch between placenta and plasma samples may be an important reason. In our study, we found the significant correlation in snoRNAs. The snoRNAs were considered to belong to the abundant small non-coding RNA (sncRNA) species. The sncRNA molecules can across the placenta barrier and then be discharged into the maternal circulation, on account of their stable structure and small size [52]. Thus, these sncRNAs could possibly consider to be prime candidates for placental and pregnancy diagnostic, and this potential was reflected again in our study. Up to date, researches have not treated the specific roles within the placenta and pregnancy of sncRNAs in much detail. In total, the contribution of sncRNAs is still an area worth exploring.

The novel cfRNA marker *ARHGEF28* validated by RT-qPCR on independent samples showed the increased expression level in maternal plasma during pregnancy and was significantly associated with increased risk of PTB. As far as we know, characterization of *ARHGEF28* was measured in the prediction of PTB firstly in our study. Previous reports demonstrated the role of *ARHGEF28* in modulating neuronal function or maintenance, and the formation of *ARHGEF28* aggregates is involved in the pathogenesis of motor neuron disease [53, 54]. Accumulating evidence has been pointed out that PTB may leave the nervous vulnerable to dysfunction [55–58]. These findings perhaps imply the *ARHGEF28* involves a role in neuron function for PTB children. We subsequently tackled the issue of predicting PTB from plasma samples collected while women were asymptomatic before 37 weeks of gestation. Collectively, the machine learning models based on *ARHGEF28* have shown excellent performance in predicting PTB, especially late PTB.

To our knowledge, this is the first study to generate cfRNA profiles as PTB biomarkers using maternal plasma in combination with RNA expression from the placenta. Analysis based on the framework proposed by us, we obtained a more comprehensive understanding of the mechanism about PTB and the regulatory role of RNA in PTB. Consistent with the previous pattern of analysis [36, 59], our profiling is based on a sample of different population cohorts which may facilitate further validation of selected biomarkers. In particular, we focused on the predictive performance of the validated markers for late PTB. We noted that late preterm infants with an increased birth rate composed now 75% of all preterm infants [60]. It is still an important public health issue. Although the validation study specifically included late PTB pregnancies, we were not assuming that late PTB was an initial independent event. In other words, discovery of potential markers that can be used to predict late PTB unaffected by previously proposed hypothetical mechanisms. As the etiology of PTB is not fully definitive, the clinical support decision model is crucial in helping doctors provide early intervention for women at high risk of PTB [61]. In this setting, we tested to garner a predictive model combining cfRNA markers and clinical factors associated with PTB, which provide a novel strategy for the development of PTB prediction model with clinical benefit. Our work further supports the application of non-invasive blood testing techniques for monitoring the risk of PTB, nevertheless, it is limited by several factors. At first, we collected placenta and plasma samples from different datasets and were not paired between two sample types, which may have led to the heterogeneity in our results and presented bias in correlation analysis. The small sample size in our study may weaken the reliability of the predictive model for the heterogeneity are more pronounced. Although we attempted to demonstrate that PTB was the only factor contributing to the increasing trend in *ARHGEF28* by selecting plasma samples collected at similar gestation weeks for additional analysis and comparison, it may be an inadequate clarification also due to the small sample size. Subsequent cohort studies on a large scale are required to resolve these concerns. In addition, PTB can be classified into three clinical subtypes including spontaneous preterm birth with intact fetal membranes, preterm premature rupture of membranes (PROM) before the onset of labor, and medically indicated preterm birth [62]. Each subtype of PTB may cause by differ pathways [63]. We were lack of adequately performing specific analysis of each subtype of PTB respectively in this study, which leads to a poor cognition of the etiological mechanisms of PTB and limits the development of predictive markers for specific subtypes. Among the RT-qPCR experiment, we found that the expression level of *ARHGEF28* was significantly increased in PTB plasma, suggesting aberrant expression of *ARHGEF28* may be related to the occurrence of PTB. However, there is no clear validation in placental tissues for the gene *ARHGEF28*. The examination of placental pathology was considered to be the major phenotype in an assessment of PTB and provided important insight into subtypes of PTB [64, 65]. We failed to extract the histopathological data of these placental specimens in this cohort study, which limited understanding of pathological features of the preterm placenta. Integration of other omics data and consideration of other clinical factors of PTB may also generate a more robust RNA profile that reveals the signature of PTB in future studies.

In summary, this study provided molecular-level evidence that RNA expression regulation is relevant in the placenta and maternal plasma in a comparison of preterm and term. The identification of novel biomarkers from plasma for preterm birth revealed the ability of cfRNA to identify the risk of PTB in advance. Combined analysis of different transcriptomic profiles of PTB can contribute to a deep mechanistic understanding of early parturition, which provides a wider perspective into the efficiency of clinical non-invasive diagnostic methods.

## Supplementary Information

**Additional file 1: Table S1.** Maternal characteristics of participants in this study involved in RT-qPCR. **Table S2.** Gene primer sequences used in RT-qPCR. **Figure S1.** Principal component analysis (PCA) for sample clustering using RNA-seq data. **Figure S2.** Comparison of plasma and placental transcriptome analysis. **Figure S3.** Complexity of RNA regulatory molecules biotype. **Figure S4.** Construction of the PPI network. **Figure S5.** The key genes in plasma and placental PPI network. **Figure S6.** Comparison of the relative expression levels of *ARHGEF28*. **Figure S7.** Predictive models for preterm birth combined with clinical factors. **Figure S8.** The exploration of relatedness between clinical factors for preterm birth and the expression level of *ARHGEF28*. **Figure S9.** The exploration of relatedness between clinical factors for preterm birth and gestational age.

**Additional file 2:** Functional annotation of hub genes in plasma and placental group.

## Data Availability

RNA sequencing data have been deposited in the Sequence Read Archive (SRA) under study accession number SRP410951.
