# Supplementary material for: Identification of novel cell-free RNAs in maternal plasma as preterm biomarkers in combination with placental RNA profiles
Source: J Transl Med. 2023 Apr 12;21:256. doi: 10.1186/s12967-023-04083-w (PMC10100253; doi:10.1186/s12967-023-04083-w)
Supplement: Supplementary file 1 — Additional file 1: Table S1. Maternal characteristics of participants in this study involved in RT-qPCR. Table S2. Gene primer sequences used in RT-qPCR. Figure S1. Principal component analysis (PCA) for sample clustering using RNA-seq data. Figure S2. Comparison of plasma and placental transcriptome analysis. Figure S3. Complexity of RNA regulatory molecules biotype. Figure S4. Construction of the PPI network. Figure S5. The key genes in plasma and placental PPI network. Figure S6. Comparison of the relative expression levels of ARHGEF28. Figure S7. Predictive models for preterm birth combined with clinical factors. Figure S8. The exploration of relatedness between clinical factors for preterm birth and the expression level of ARHGEF28. Figure S9. The exploration of relatedness between clinical factors for preterm birth and gestational age. [file 12967_2023_4083_MOESM1_ESM.docx]

**ADDITIONAL FILE 1**

**Additional figures and tables**

**Table S1 Maternal characteristics of participants in this study involved in RT-qPCR.**

|  | Preterm birth  (n=24) | Full term  (n=17) |
| --- | --- | --- |
| Maternal age (years) | 30.3±3.5 | 30.1±5.0 |
| Sample GA (weeks) | 34.7±1.9 | 39.7±0.8 |
| Nulliparous (%) | 54.2 | 35.3 |
| Male fetus (%) | 47.1 | 41.0 |
| Maternal BMI (kg/m^2^) | 26.2±3.8 | 27.4±3.6 |

**Table S2 Gene primer sequences used in RT-qPCR.**

| **Gene** | **Forward Primer** | **Reverse Primer** |
| --- | --- | --- |
| CA2 | ATCGACACTCATACAGCCAAGT | AAAGCATGACCATTGTTGAGGA |
| VTRNA1-2 | CTGGCTTTAGCTCAGCGGTT | AAAGAGCTGGAAAGCACCCG |
| ARHGEF28 | CAGTGAAGTGCCTCTTTATGGAC | ACACGGTCAGCAATCACCAC |
| LIPH | AGGACACATAGACTTCTACCCG | CCTTCGTCATCGGAGGATCTC |
| PAX5 | CCATCAGGACAGGACATGGAG | GGCAAGTTCCACTATCCTTTGG |
| CRISP3 | TATCTACAGCCTCAAAATAAGCCATGCAGAA | ATAGATGTCGGAGTTTTATTCGGTACGTCTT |
| CAMK4 | GAGAACCTCGTCCCGGATTAC | ACACAATGGATGTAGCACCCC |
| GAPDH | GAACGGGAAGCTTGTCATCAA | ATCGCCCCACTTGATTTTGG |
| ERCC96 | CAACGGTGCAATCTCAGCTA | CACGAGGATGTTCCTGTTGA |

**Fig. S1 Principal component analysis (PCA) for sample clustering using RNA-seq data.**

**
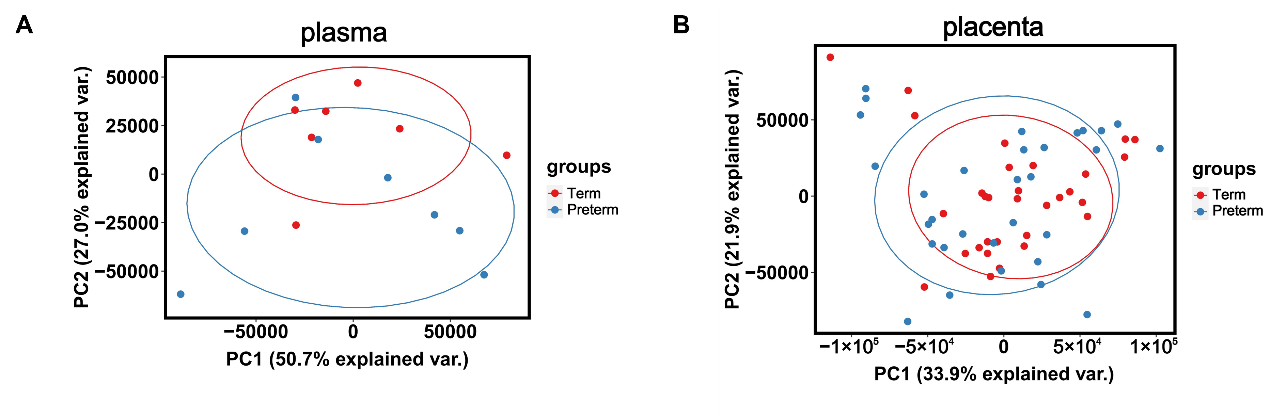
(A)** PCA for the discovery of sample clustering features was applied in plasma datasets. **(B)** PCA for the discovery of sample clustering features was applied in placenta datasets.

**Fig. S2 Comparison of plasma and placental transcriptome analysis.**


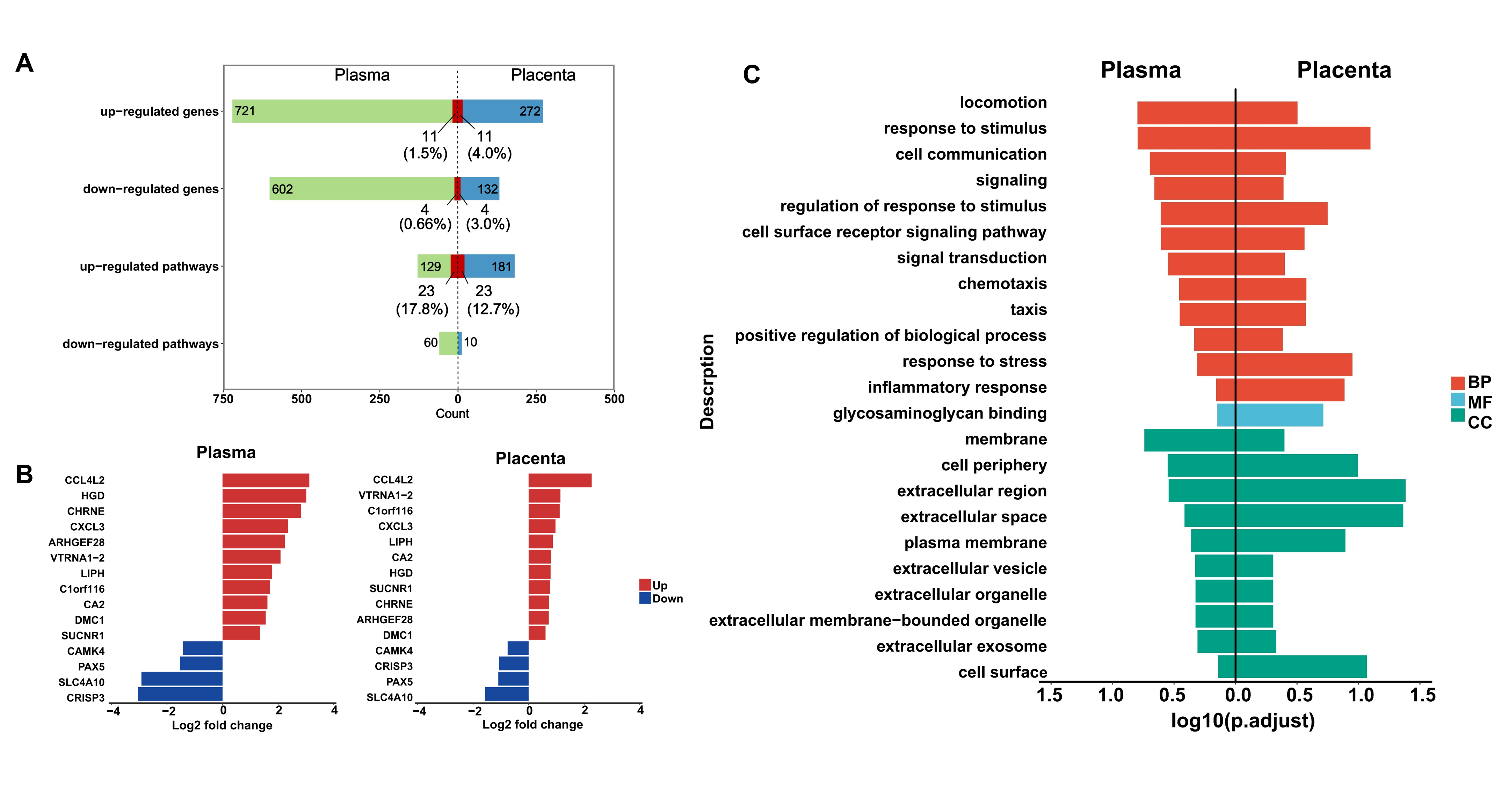


**(A)** The comparison of numbers of differential expression genes and enrichment pathway between plasma and placenta groups. Red represents overlapping portions of plasma and placenta. **(B)** The log_2_ fold change of overlapped DEGs in the plasma and placenta. **(C)** enrichment of the common pathway in the plasma and the placenta groups.

**Fig. S3 Complexity of RNA regulatory molecules biotype.**


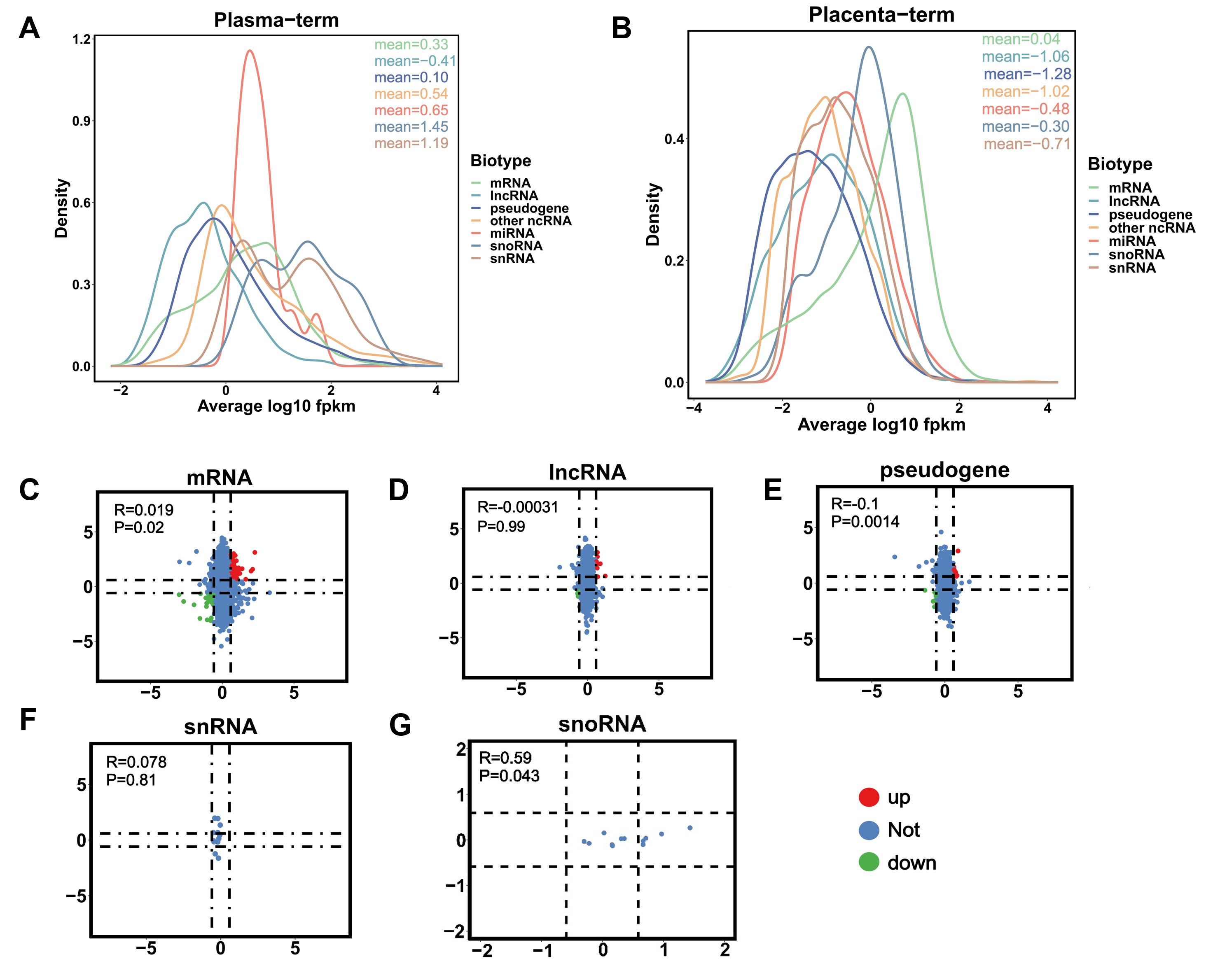


**(A-B)** Distribution of expression abundance in full term of different types of RNA compared in **(A)** plasma, **(B)** placenta. **(C-G)** Correlation of expression changes in placenta and plasma of different RNA biotypes including **(C)** mRNA, **(D)** long non-coding RNA, **(E)** pseudogene, **(F)** snRNA, and **(G)** snoRNA. x-axis: the log_2_ fold change in plasma, y-axis: the log_2_ fold change in the placenta. up: up-regulated, log_2_ fold change ≥ 0.59. down: down-regulated, log_2_ fold change≤ -0.59.

**Fig. S4 Construction of the PPI network.**

**
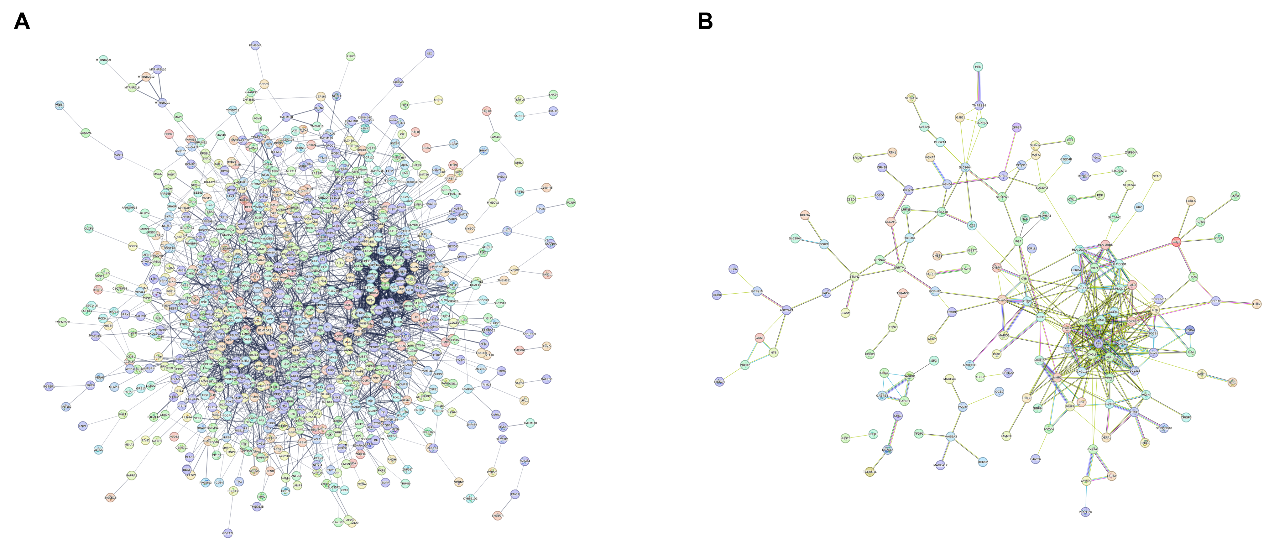
**

**(A)** The PPI network is constructed by differential expression genes from plasma. **(B)** The PPI network is constructed by differential expression genes from placenta.

**Fig. S5 The key genes in plasma and placental PPI network.**


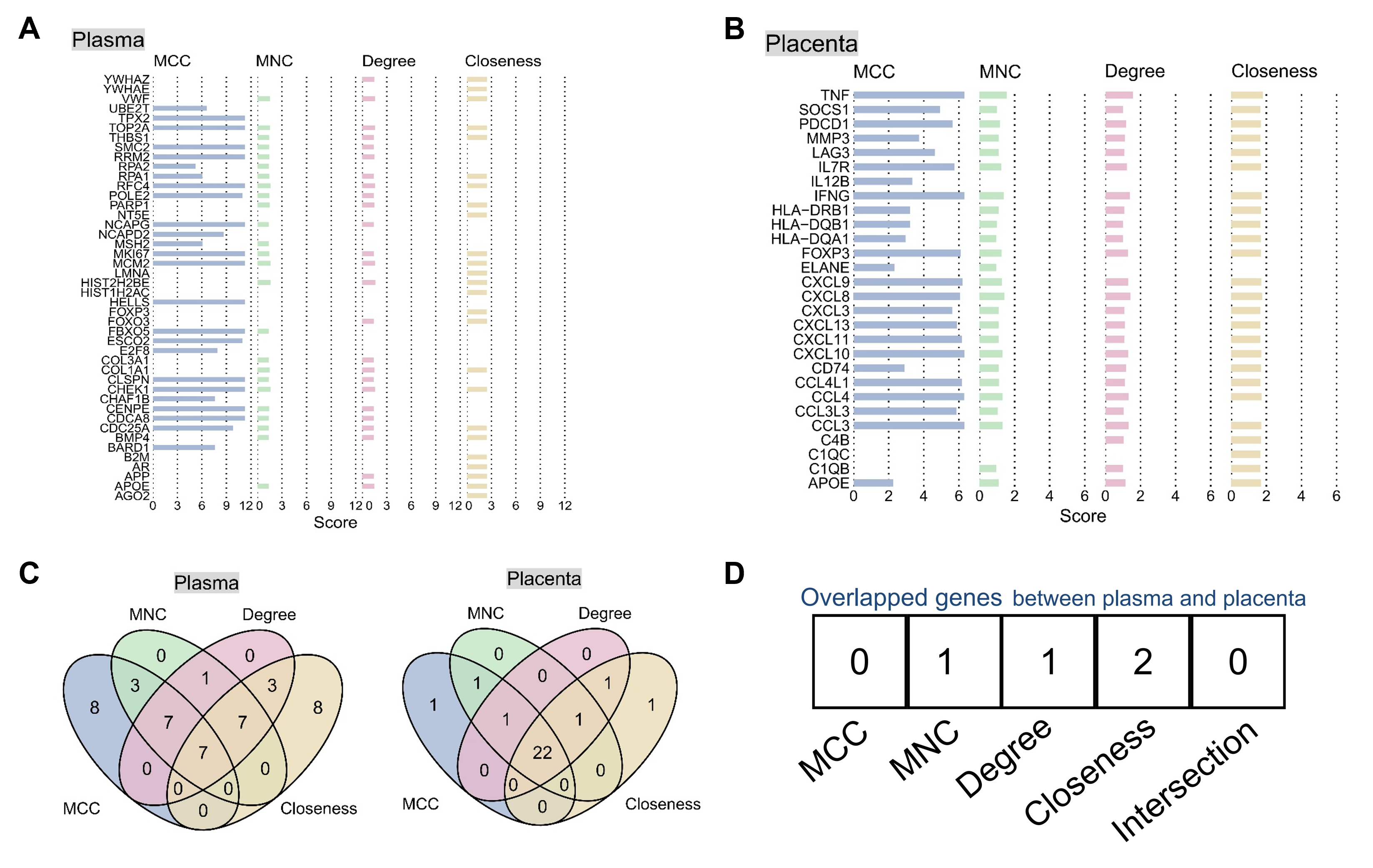


**(A)** The score of top 25 key genes in each algorithm in the PPI network constructed by differential expression genes from plasma. **(B)** The score of top 25 key genes in each algorithm in the PPI network constructed by differential expression genes from placenta. **(C)** Venn diagram of the identified hub genes. **(D)** Comparison of the overlapped genes of the different results between plasma and placenta.

**Fig. S6 Comparison of the relative expression levels of *ARHGEF28***

**
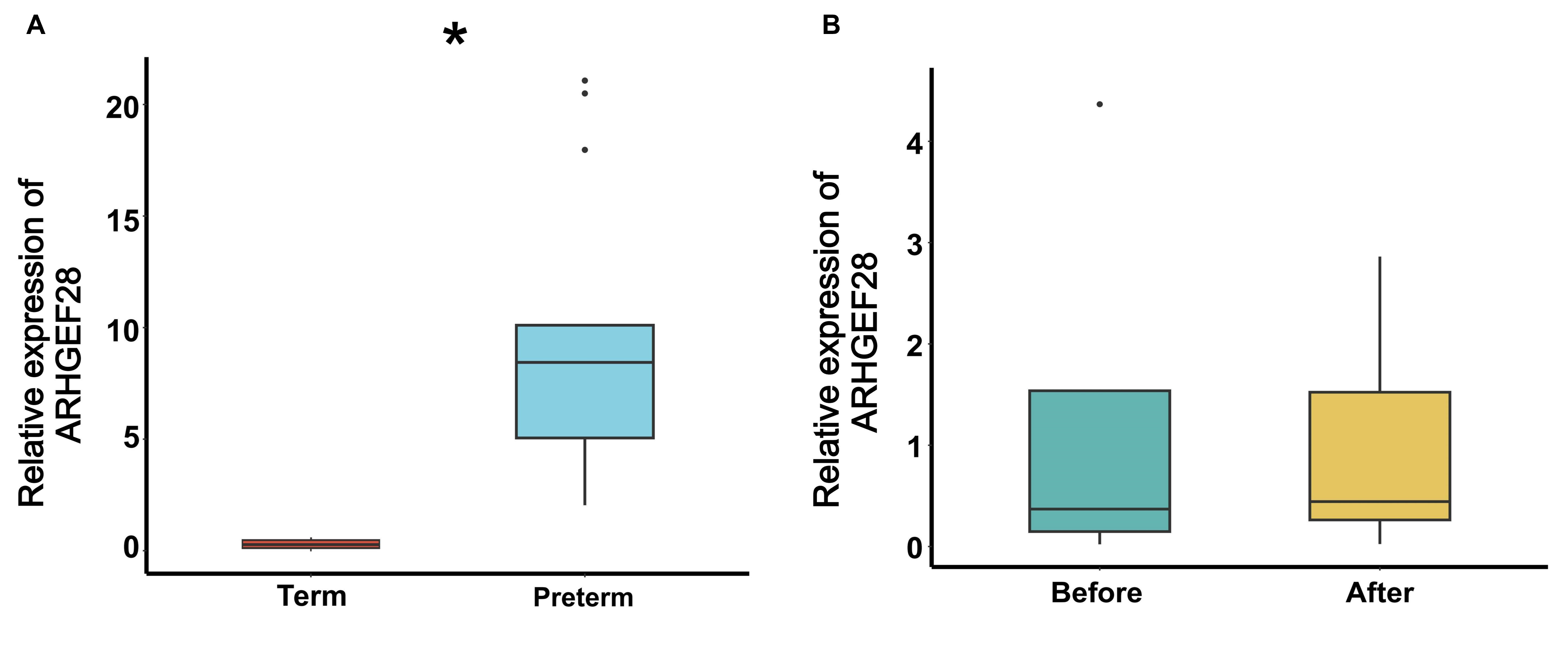
**

**(A)** The relative expression level of *ARHGEF28* between the term and preterm samples collected at 35±1 weeks. **(B)** The relative expression level of *ARHGEF28* between the samples collected before 37 weeks and after 37 weeks. *p-value<0.05

**Fig. S7 Predictive models for preterm birth combined with clinical factors.**

**
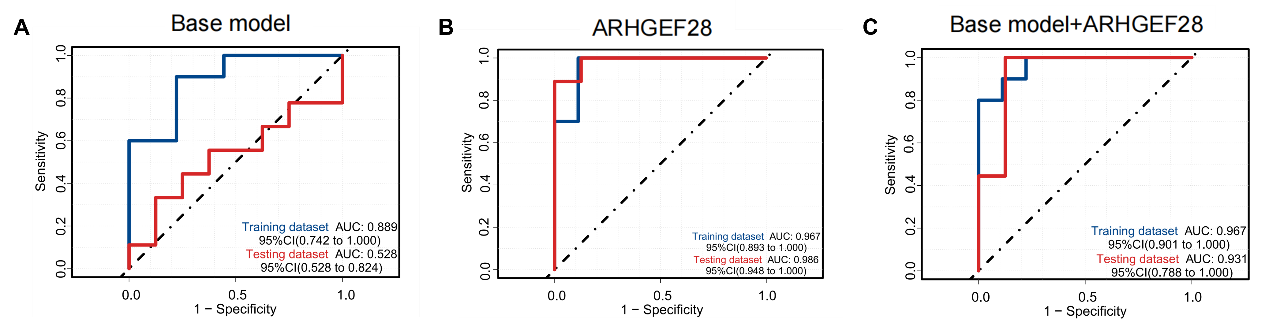
**

**(A)** Development of a base model for predicting preterm birth using clinical factors. **(B)** Development of a model for predicting preterm birth using the cfRNA *ARHGEF28* in 36 samples. **(C)** The six risk factors and *ARHGEF28* were combined to development of a model for predicting preterm birth.

**Fig. S8 The exploration of relatedness between clinical factors for preterm birth and the expression level of *ARHGEF28*.**

**
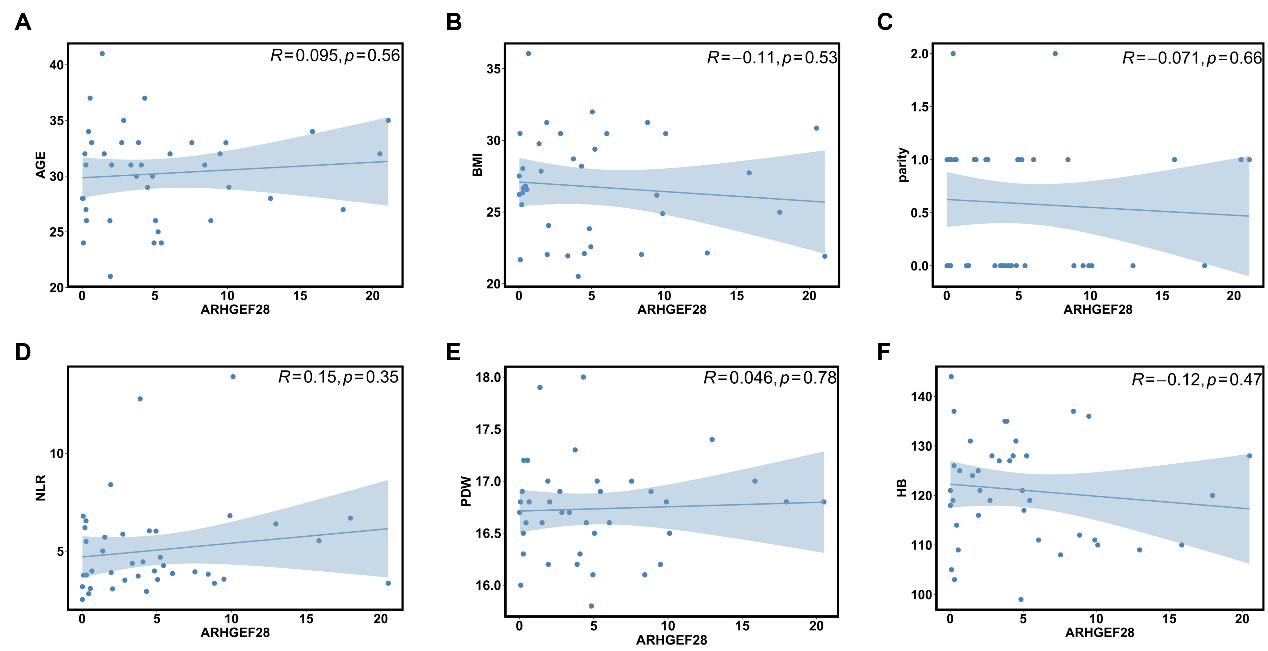
**

**(A)** Scatter plot of the *ARHGEF28* expression and maternal age. **(B)** Scatter plot of the *ARHGEF28* expression and maternal BMI. **(C)** Scatter plot of the *ARHGEF28* expression and parity. **(D)** Scatter plot of the *ARHGEF28* expression and maternal NLR level. **(E)** Scatter plot of the *ARHGEF28* expression and maternal PDW level. **(F)** Scatter plot of the *ARHGEF28* expression and maternal HB level.

**Fig. S9 The exploration of relatedness between clinical factors for preterm birth and gestational age.**

**
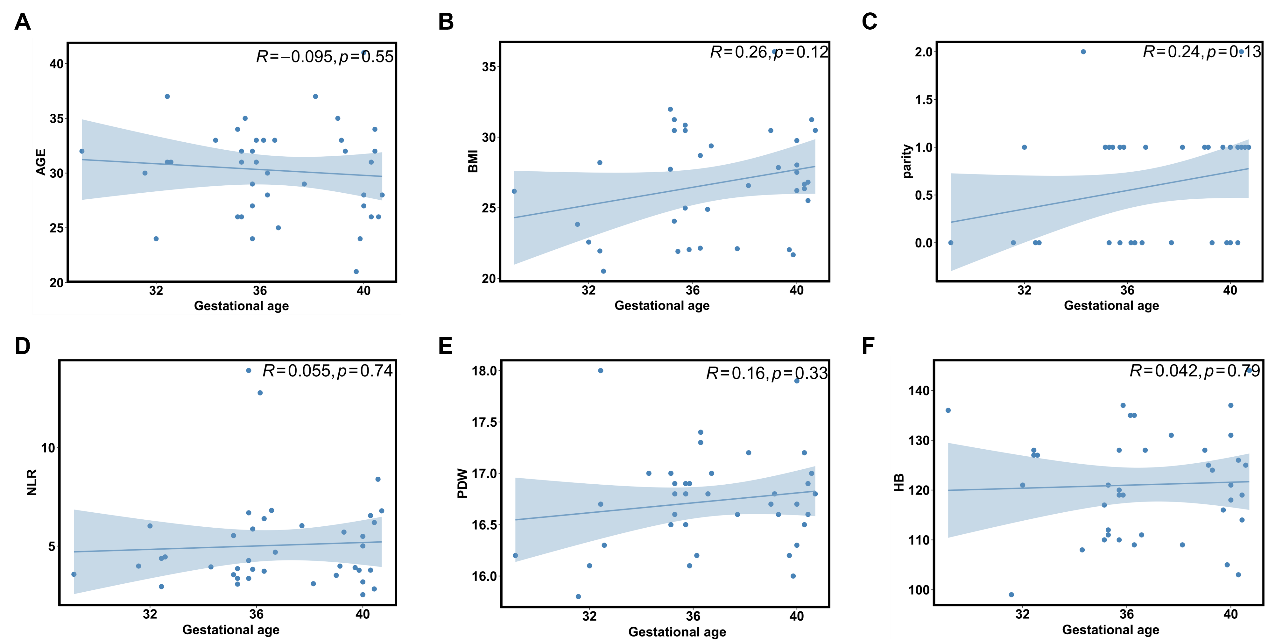
**

**(A)** Scatter plot of the temporal changes in gestational age and maternal age. **(B)** Scatter plot of the temporal changes in gestational age and maternal BMI. **(C)** Scatter plot of the temporal changes in gestational age and parity. **(D)** Scatter plot of the temporal changes in gestational age and maternal NLR level. **(E)** Scatter plot of the temporal changes in gestational age and maternal PDW level. **(F)** scatter plot of the temporal changes in gestational age and maternal HB level.
